# *Klebsiella pneumoniae* manipulates human macrophages to acquire iron

**DOI:** 10.3389/fmicb.2023.1223113

**Published:** 2023-08-11

**Authors:** Philipp Grubwieser, Richard Hilbe, Clemens Michael Gehrer, Manuel Grander, Natascha Brigo, Alexander Hoffmann, Markus Seifert, Sylvia Berger, Igor Theurl, Manfred Nairz, Günter Weiss

**Affiliations:** ^1^Department of Internal Medicine II, Infectious Diseases, Immunology, Rheumatology, Pulmonology, Medical University of Innsbruck, Innsbruck, Austria; ^2^Christian Doppler Laboratory for Iron Metabolism and Anemia Research, Medical University of Innsbruck, Innsbruck, Austria

**Keywords:** macrophages, *Klebsiella pneumoniae*, immune metabolism, iron, transferrin, transferrin receptor, interleukin-10

## Abstract

**Background:**

*Klebsiella pneumoniae* (KP) is a major cause of hospital-acquired infections, such as pneumonia. Moreover, it is classified as a pathogen of concern due to sprawling anti-microbial resistance. During infection, the gram-negative pathogen is capable of establishing an intracellular niche in macrophages by altering cellular metabolism. One factor critically affecting the host-pathogen interaction is the availability of essential nutrients, like iron, which is required for KP to proliferate but which also modulates anti-microbial immune effector pathways. We hypothesized, that KP manipulates macrophage iron homeostasis to acquire this crucial nutrient for sustained proliferation.

**Methods:**

We applied an *in-vitro* infection model, in which human macrophage-like PMA-differentiated THP1 cells were infected with KP (strain ATCC 43816). During a 24-h course of infection, we quantified the number of intracellular bacteria *via* serial plating of cell lysates and evaluated the effects of different stimuli on intracellular bacterial numbers and iron acquisition. Furthermore, we analyzed host and pathogen specific gene and protein expression of key iron metabolism molecules.

**Results:**

Viable bacteria are recovered from macrophage cell lysates during the course of infection, indicative of persistence of bacteria within host cells and inefficient pathogen clearing by macrophages. Strikingly, following KP infection macrophages strongly induce the expression of the main cellular iron importer transferrin-receptor-1 (TFR1). Accordingly, intracellular KP proliferation is further augmented by the addition of iron loaded transferrin. The induction of TFR1 is mediated *via* the STAT-6-IL-10 axis, and pharmacological inhibition of this pathway reduces macrophage iron uptake, elicits bacterial iron starvation, and decreases bacterial survival.

**Conclusion:**

Our results suggest, that KP manipulates macrophage iron metabolism to acquire iron once confined inside the host cell and enforces intracellular bacterial persistence. This is facilitated by microbial mediated induction of TFR1 *via* the STAT-6-IL-10 axis. Mechanistic insights into immune metabolism will provide opportunities for the development of novel antimicrobial therapies.

## 1. Introduction

Bacterial infections remain a leading cause of death and a source of health loss, globally. A recent global burden of disease study defined five bacterial pathogens as responsible for over 50% of deaths: *Staphylococcus aureus*, *Escherichia coli*, *Streptococcus pneumoniae*, *Klebsiella pneumoniae* (KP), and *Pseudomonas aeruginosa* (Ikuta et al., 2022). Specifically, KP has evolved to pose a major clinical and public health threat due to sprawling antimicrobial resistance (Dong et al., 2022). KP is a gram-negative, rod shaped bacterium of the *Enterobacteriaceae* family, which is typically found in the microbiome of the skin, gastrointestinal and upper respiratory tract. The opportunistic pathogen causes a broad range of infections, including respiratory tract, urinary tract and systemic infections. Especially in healthcare, KP is a predominant cause of nosocomial infections, as approximately 50% of all KP infections are healthcare-associated (Kang et al., 2006; Jean et al., 2020; Le et al., 2021).

Numerous virulence factors contribute to the pathogenicity of KP, allowing this pathogen to evade host immune functions. Capsular polysaccharide (CPS), lipopolysaccharide (LPS), fimbriae, as well as redundant iron uptake systems are recognized as the major pathogenic factors (Huynh et al., 2017; Riwu et al., 2022). Iron is an essential nutrient KP depends on to sustain growth, as the metal is implicated in bacterial metabolism, DNA synthesis and detoxification of reactive oxygen species (Andrews et al., 2003). Bacterial iron acquisition represents an area of extensive research. Exemplary for this, a novel antimicrobial drug, cefiderocol, exploits bacterial iron demand as a route of entry and is highly effective even against carbapenem-resistant isolates (Bonomo, 2019; Rodríguez and González-Bello, 2023). During infection, the pathogen relies on iron acquisition from its microenvironment, which is facilitated primarily by ferrous iron transporters and siderophores, which are high-affinity iron chelating molecules (Andrews et al., 2003). The expression of the bacterial iron uptake systems are tightly regulated by iron availability and linked to pathogenicity (Frawley and Fang, 2014; Muselius et al., 2020). Interestingly, such regulation patterns are detectable in clinical isolates, and a reduction in bacterial iron uptake systems during growth in iron-rich conditions may negatively affect the efficacy of cefiderocol, which is normally taken up *via* the siderophore uptake system (Daoud et al., 2022; Escalante et al., 2023).

In turn, the host has evolved mechanisms to antagonize bacterial iron acquisition on multiple levels. This innate immune strategy is referred to as nutritional immunity, primarily involving macrophages, but also in non-professional immune cells (Weinberg, 1975; Deriu et al., 2013; Núñez et al., 2018; Nairz and Weiss, 2020; Grubwieser et al., 2022). The significance of iron availability in the context of infection has been demonstrated for multiple pathogens in *in-vitro* and *in-vivo* models (Nairz et al., 2008; Drakesmith and Prentice, 2012; Cassat and Skaar, 2013; Lin et al., 2014; Iatsenko et al., 2020; Hoffmann et al., 2021; Sargun et al., 2021; Grander et al., 2022).

Macrophages belong to the first line of innate immune defense, and play an important role in the containment and clearance of KP (Broug-Holub et al., 1997; Cheung et al., 2000). Although typically regarded as an extracellular pathogen, during infection, KP has been observed inside macrophages and epithelial cells *in-vitro* and *in-vivo* (de Astorza et al., 2004; Willingham et al., 2009; Chuquimia et al., 2012; Hsu et al., 2015). Furthermore, once confined inside macrophages, KP manipulates phagosome maturation and thus establishes an intracellular niche for survival (Cano et al., 2015). Only recently, evidence has emerged that KP has the ability to manipulate macrophage metabolism, which results in a more permissive environment for the pathogen (Dumigan et al., 2022; Feriotti et al., 2022; Wong Fok Lung et al., 2022). Although, several mechanisms are involved in that process, studies implicate a disproportionately high interleukin (IL)-10 secretion to be associated with host tolerance to infection. IL-10 is an anti-inflammatory cytokine which inhibits several pro-inflammatory and anti-microbial immune effector pathways in macrophages (Bogdan and Nathan, 1993). As this anti-inflammatory cytokine directly affects iron metabolism in macrophages (Tilg et al., 2002; Ludwiczek et al., 2003), we hypothesized that IL-10 induction by KP manipulates iron metabolism in macrophages, and increases the bacterial access to this essential nutrient.

## 2. Materials and methods

### 2.1. Cell and bacterial culture

THP-1 human monocytic cells (DMSZ ACC 16) were propagated in RPMI (PAN-Biotech) containing 10% FBS (PAN-Biotech), 1% Penicillin/Streptomycin (Lonza), 1% NEAAs (Gibco) and 2% Na-Pyruvat (Gibco). Before infection experiments, 5 × 10^5^ cells were seeded onto 12-well plates and differentiated with 20 ng/mL Phorbol-12-myristate-13-acetate (PMA) for 48 h. After differentiation, macrophage-like cells were washed with PBS and the medium was changed to RPMI containing 1% FBS and no antibiotics.

*Klebsiella pneumoniae* (ATCC 43816) was grown from overnight cultures under sterile conditions in LB Broth (Sigma-Aldrich) to mid logarithmic growth phase [optical density 600 nm (OD_600_) 0.4–0.6]. Bacterial counts were determined before each experiment using a cell counter and analyzer (CASY, 45 μm capillary, OLS OMNI Life Science).

Bacteria constitutively expressing the fluorescent protein Ypet were previously described (Grubwieser et al., 2022). For the KP iron sensing strain, bacteria were made electro-competent using glycerol/mannitol density step centrifugation, as described in an established protocol (Warren, 2011). The plasmid pAH05 with genes encoding for blue fluorescent protein (BFP) downstream of the iron sensitive RhyB2 promotor, green fluorescent protein (GFP) downstream of the SodB promotor, and mCherry downstream of the constitutively active PybaJ promoter was electroporated into KP (NCBI: OQ979407).

For experiments with heat-inactivated KP, bacteria were incubated at 70°C for 20 min. For inactivation with gentamicin, KP was incubated in 50 μg/mL gentamicin (Life Technologies) for 30 min. After inactivation, bacteria were plated on LB plates to confirm the absence of viable bacteria.

For the bacterial growth assay, bacteria were diluted to OD_600_ of 0.005 in 50 μL cell culture medium in sterile 96-well plates (Greiner bio-one), and directly afterwards incubated in an automated microplate reader (Spark, TECAN) at 37°C, 5% CO_2_. OD_600_ and fluorescence intensity were measured every 15 min after a double orbital shaking interval for a total of 14 h.

### 2.2. *In-vitro* infection

To quantify intracellular bacteria, we applied a gentamicin-protection assay. Cells were infected with KP at a multiplicity of infection (MOI) of 10. After 1 h of active infection, cells were washed thrice with PBS containing gentamicin (Life Technologies, 50 μg/mL) and incubated in a fresh medium containing 1% FBS and gentamicin (25 μg/mL). This treatment prevents bacterial overgrowth, and as gentamicin exclusively eliminates extracellular bacteria, allows further incubation of macrophages containing intracellular viable bacteria. Uninfected controls were treated with identical washing and incubation steps. To quantify the number of viable intracellular pathogens, cells were washed thrice again in PBS and afterwards lysed in 0.5% sodium deoxycholic acid (Sigma-Aldrich) at indicated time points. Cell lysates were then serially diluted and plated onto LB-plates. After overnight incubation at 37°C, colony-forming units (CFUs) were quantified.

When cells were treated with inactivated bacteria, an MOI of 50 was used to account for the lack of bacterial growth compared to the viable bacteria during the 1 h active infection phase. After 1 h of stimulation, cells were washed and incubated in a fresh medium containing 1% FBS and gentamicin, equal to cells infected with viable bacteria.

Where indicated, cells were stimulated during the gentamicin-protected infection phase (directly after washing and addition of gentamicin-containing medium) with either 50 μg/mL iron-loaded transferrin (TF, Sigma-Aldrich), 1 μM STAT-6 inhibitor AS1517499 (MedChem Express), 40 μg/mL anti-human IL-10 blocking antibody (Biolegend) or 40 ng/mL recombinant human IL-10 (Peprotech).

### 2.3. Fluorescence microscopy

For immune fluorescence imaging, cells were seeded and differentiated in 8-well chamber slides (Falcon) and infected with fluorescent (Ypet) bacteria. Afterwards, cells in chamber slides were washed with PBS and fixed with 4% paraformaldehyde for 10 min. Cells were then permeabilized with 0.5% saponin (Sigma-Aldrich) for 30 min, stained with Alexa-flour-647-labeled CD71 (TFR1) antibody (Biolegend) for 2 h, and 4′,6-diamidino-2-phenylindole (DAPI, BioLegend) for 30 min. After staining, samples were mounted with Faramount Mounting Medium (Dako). Fluorescence microscopy was performed immediately after sample preparation using a VS120-S6 fluorescence microscope (Olympus). Images were captured with a 40-x objective using 387/440 nm (DAPI), 485/525 nm (Ypet), and 650/684 nm (Alexa-flour-647) lasers and filters, under identical exposure times for every sample. Fluorescence intensity of Alexa-flour-647 was determined with the CellSense (Olympus) program for at least 100 cells per sample.

### 2.4. Quantitative real-time PCR

The quantitative real-time PCR was carried out as described elsewhere (Hoffmann et al., 2021). In short, total sample RNA was isolated using acid guanidinium thiocyanate-phenol-chloroform extraction with peqGOLD Tri-Fast™ (Peqlab). For reverse transcription, 2 μg RNA, random hexamer primers (200 ng/μL) (Roche), dNTPs (10 mM) (GE Healthcare LifeSciences) 20 U RNasin (Promega) and 200 U M-MLV reverse transcriptase (Invitrogen) in first-strand buffer (Invitrogen) were used. Ssofast Probes Supermix and Ssofast EvaGreen Supermix (Bio-Rad Laboratories GmbH) were used according to the manufacturer’s instructions. Real-time PCR reactions were performed on QuantStudio 3 and 5 real-time PCR systems (Thermo Fisher Scientific). Gene expression was normalized using the ΔΔct method. Tubulin (TUB) and ornithine decarboxylase antizyme 1 (OAZ1) were used as reference transcripts. TaqMan PCR primers used in this study are listed in Table 1.

**Table 1:** TaqMan PCR primers used in this study (all 5′ → 3′).

Target	Forward primer	Reverse primer	Probe
TUB	TCCTTCAACACCTTCTTCAGTGAGACG	GGTGCCAGTGCGAACTTCATCA	ATGTGCCCCGGGCAGTGTTTGTAGACTTG
OAZ1	GGATCCTCAATAGCCACTGC	TACAGCAGTGGAGGGAGACC	TGGATGGTGGCGCTGGGTTTATC
TFR1	TCCCAGCAGTTTCTTTCTGTTTT	CTCAATCAGTTCCTTATAGGTGTCCA	CGAGGACACAGATTATCCTTATTTGGGTACCACC
IL-10	ATGCCCCAAGCTGAGAAC	GCCTTGCTCTTGTTTTCACAG	

### 2.5. Western blot

Protein extraction and Western blotting were performed as described previously (Hoffmann et al., 2021). The following antibodies were used: a mouse TFR1 antibody (1,1,000; Sigma Cat# SAB4300398), a rabbit FT antibody (1,500; Sigma, F5012), and a rabbit actin antibody (1,500, Sigma Cat# A2066). Appropriate HRP-conjugated secondary antibodies (1,2000, anti-rabbit, Dako Cat# P0399; 1:4000, anti-mouse; Dako Cat# P0447) were used. For quantification, densitometry data were acquired on a ChemiDoc Touch Imaging System (Bio-Rad) and analyzed with Image Lab 5.2.1. (Bio-Rad Laboratories GmbH).

### 2.6. ELISA

A human IL-10 ELISA Set (BD Biosciences) was used to measure secreted IL-10 levels in cell culture supernatants, according to the manufacturer’s protocol.

### 2.7. Measurement of ferrous iron by FerroOrange fluorescence

For evaluation of free cellular iron, cells were washed with PBS and stained with 250 μM FerroOrange (FO, Dojindo) for 30 min. FO is a fluorescent probe that detects intracellular Fe^2+^ ions with high specificity and does not react with chelated iron or other bivalent metals (Hirayama et al., 2020). Subsequently, fluorescence intensity (540 nm/585 nm) was determined using a multimode microplate reader (Spark, TECAN) at 16 different localizations in each well.

### 2.8. Flow cytometry

To assess the fluorescence of intracellular bacteria, flow cytometry was applied. Cells were infected with KP containing the pAH05 plasmid and underwent the gentamicin protection assay as described in 2.2. After 24 h, cells were harvested in FACS-buffer containing 0.5% FBS and 2 mM ethylenediaminetetraacetic acid (EDTA) and immediately subject to flow cytometry analysis. Data were acquired on a CytoFLEX S (Beckman Coulter) and subsequently analyzed with FlowJo 10.7.0 software (Beckton Dickinson). After gating for cells and single cells, infected cells were identified as mCherry+. In infected cells, BFP MFI was acquired to evaluate the status of bacterial iron starvation. Gating of infected cells is depicted in Figure 4.

### 2.9. Statistical analysis

Statistical analysis was carried out using GraphPad Prism version 9.1 for Windows and Mac (GraphPad Software). Data are presented as mean with 95% CI, SD or SEM as dispersion characteristic. Significant differences between groups were determined using unpaired t test or ANOVA with *post-hoc* analysis. Multiple comparisons were adjusted using Tukey’s or Holm-Šídák’s methods. For non-normal distributed data, as evaluated by Shapiro–Wilk-test, a Kruskal-Wallis test with Dunn’s multiple comparisons test was performed. *p* < 0.05 was used as the significance threshold.

## 3. Results

### 3.1. Klebsiella infects human macrophages and induces TFR1 expression

**Figure 1:**
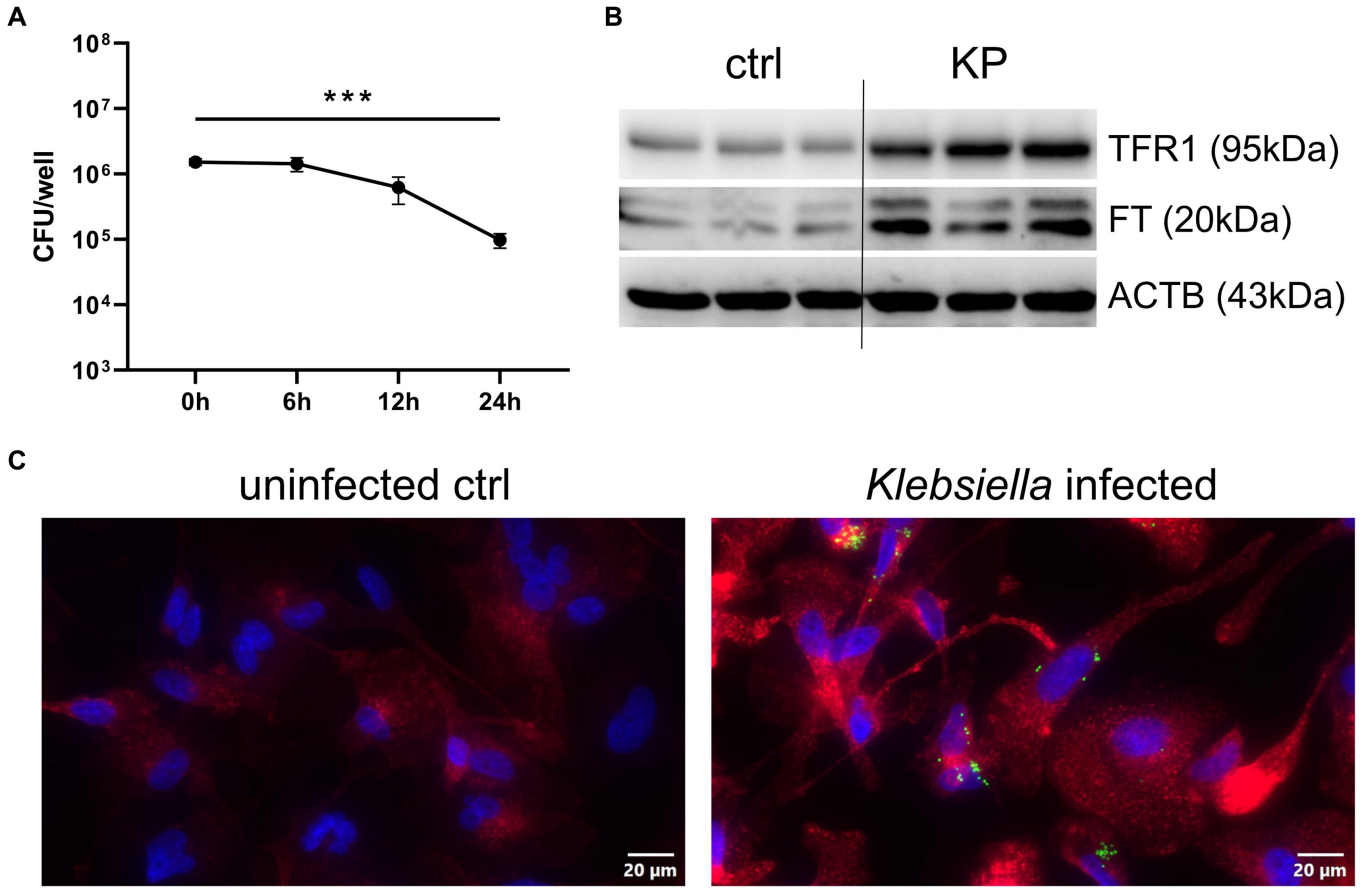
Klebsiella infects human macrophages and induces TFR1. **(A)** Quantification of intracellular bacteria during the 24 h course of infection. PMA-differentiated THP-1 cells were infected with KP for 1 h at a MOI of 10 and were lysed directly after incubation phase (0 h time point) or at noted time intervals of gentamicin-protected, intracellular infection. Bacteria-containing lysates were serially diluted and plated onto LB-agar plates for CFU quantification. Data are shown as mean CFU/well lysate ±95% CI of three separate experiments. **(B)** Western blot of the iron uptake protein TFR1 and the iron storage protein FT in KP infected cells. Representative blot after 24 h of infection of three separate experiments. **(C)** Immune fluorescence imaging revealing intracellular bacteria and induction of TFR1 in infected cells after 24 h of infection. Representative images, showing Ypet expressing bacteria (green rods) in cells stained for TFR1 (red) and nuclei (blue) at 400x magnification with a 20 μM scale bar. ^***^ denotes *p* < 0.001 for *post-hoc* statistical testing. KP, *Klebsiella pneumoniae*; ctrl, control; CFU, colony forming units; TFR1, transferrin-receptor-1; FT, ferritin; ACTB, β-actin.

To study the KP-macrophage interaction, we infected PMA differentiated THP1 cells with viable bacteria for 1 h. We then applied a gentamicin-protection assay, which eliminates extracellular bacteria but preserved viable bacteria in the intracellular space of macrophages over the course of 24 h (Figure 1A). Although bacterial numbers declined over time, macrophages were unable to fully eliminate the pathogen during the 24 h infection phase. This is indicative of bacterial resistance in this host-pathogen interaction, in which numerous host factors influence the outcome, including cellular iron metabolism. To gain insight into alterations of macrophage iron homeostasis, we performed western blots of critical iron proteins in uninfected controls and KP infected cells after 24 h. Interestingly, protein levels of the main cellular iron importer, the transferrin-receptor-1 (TFR1), were strongly induced in KP infected cells (Figure 1B; Supplementary Figure S1). A time course analysis of TFR1 protein levels revealed that induction was already present after 12 h of infection, but was most pronounced after 24 h of infection (Supplementary Figure S2). Accordingly, the iron storage protein ferritin (FT) was upregulated in KP infected cells. To shed light on this host-pathogen interaction, we applied immunofluorescence using bacteria that were transformed to constitutively express the Ypet fluorescence protein (Figure 1C). Intracellular bacteria were visible in infected macrophages (green, rod shaped) and the induction of TFR1 (red) was again confirmed. TFR1 expression was enhanced in both, cells containing bacteria and bystander cells, indicative of an autocrine and paracrine mechanism driving this upregulation.

Together, these results implicate that once inside the host cell, KP establishes an intracellular niche to facilitate infection, and this process is associated with higher expression of the main cellular iron uptake protein, TFR1.

### 3.2. Klebsiella induces iron uptake to establish infection

**Figure 2:**
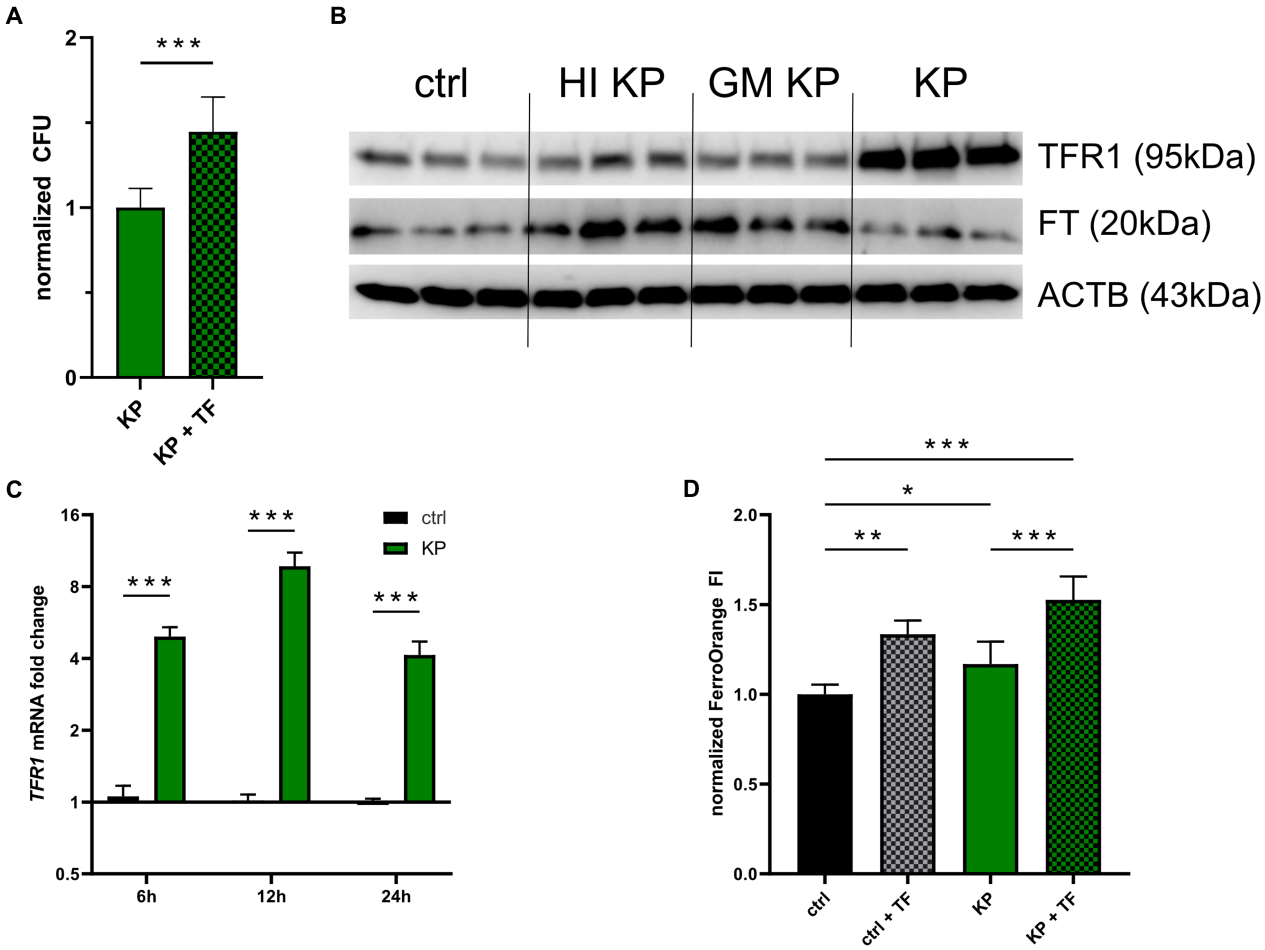
Klebsiella induces iron uptake to establish infection. **(A)** Intracellular bacteria benefit from iron loaded TF stimulation. PMA-differentiated THP-1 cells were infected with KP for 1 h at a MOI of 10 and treated with 50 μg/mL iron loaded TF during the 24 h gentamicin-protected, intracellular infection phase. Afterwards, bacteria-containing lysates were serially diluted and plated onto LB-agar plates for CFU quantification. Data shown as mean ± 95% CI of three separate experiments, normalized to control condition. **(B)** Western blot of the iron uptake protein TFR1 and the iron storage protein FT in cells treated with inactivated bacteria or infected with viable KP. Representative blot after 24 h of infection of two separate experiments. **(C)** Differential TFR1 mRNA expression in KP infected cells. Infected cells and uninfected controls were harvested at indicated time intervals of intracellular, gentamicin protected infection. Data shown as mean ± SEM of three separate experiments. **(D)** Measurement of cellular iron levels *via* the fluorescent probe FerroOrange. Cells underwent 24 h gentamicin-protected KP infection or treatment with iron loaded TF, and subsequently FI of FerroOrange was measured. Data from two separate experiments shown as mean ± SD, normalized to untreated controls. ^*^ denotes *p* < 0.05, ^**^ denotes *p* < 0.01, ^***^ denotes p < 0.001 for *post-hoc* statistical testing. KP, *Klebsiella pneumoniae*; TF, transferrin; ctrl, control; CFU, colony forming units; HI, heat-inactivated; GM, gentamicin-killed; TFR1, transferrin-receptor-1; FT, ferritin; ACTB, β-actin.

Next, we aimed to uncover whether TFR1 induction is solely a consequence of the host-response to bacterial invasion, or aids the pathogen to establish infection in macrophages. For this, we stimulated infected cells with 50 μg/mL iron-loaded transferrin (TF), to facilitate increased iron availability for KP *via* the TFR1-mediated iron uptake. Interestingly, KP infected cells treated with TF showed increased intracellular pathogen numbers compared to not TF supplemented cells after 24 h of infection (Figure 2A). This raises the possibility, that TFR1 induction is mediated by the intracellular pathogen itself, as increased cellular iron availability benefits the intracellular pathogen. Following this lead, we treated cells with either heat-inactivated, gentamicin-killed, or viable KP bacteria and subsequently analyzed TFR1 and FT protein expression (Figure 2B; Supplementary Figure S1). Strikingly, only viable bacteria were able to induce the main iron importer TFR1. Of note, FT induction was solely increased in cells treated with inactivated bacteria. This suggested that a specific factor originating from viable intracellular bacteria is responsible for TFR1 induction. In contrast, sterile pathogen associated molecular patterns from inactivated bacteria, like LPS, seem to be responsible for FT induction. Subsequently, we quantified TFR1 mRNA levels, to shed light on the specific induction mechanism (Figure 2C). Consistent with our protein data, mRNA expression of TFR1 was significantly increased in KP infected cells compared to uninfected controls over the course of infection. We then studied cellular iron accumulation employing the fluorescent probe FerroOrange (FO, Figure 2D). In cells treated with TF, significantly more iron was measured compared to untreated controls. Cells infected with KP showed significantly more FO fluorescence compared to uninfected cells. Interestingly, infected cells treated with TF showed the highest fluorescence level, being indicative for more cellular iron incorporation. Collectively, these results indicate that viable KP induces TFR1-mediated iron uptake in macrophages.

### 3.3. TFR1 induction is mediated by the STAT6-IL-10 axis

**Figure 3:**
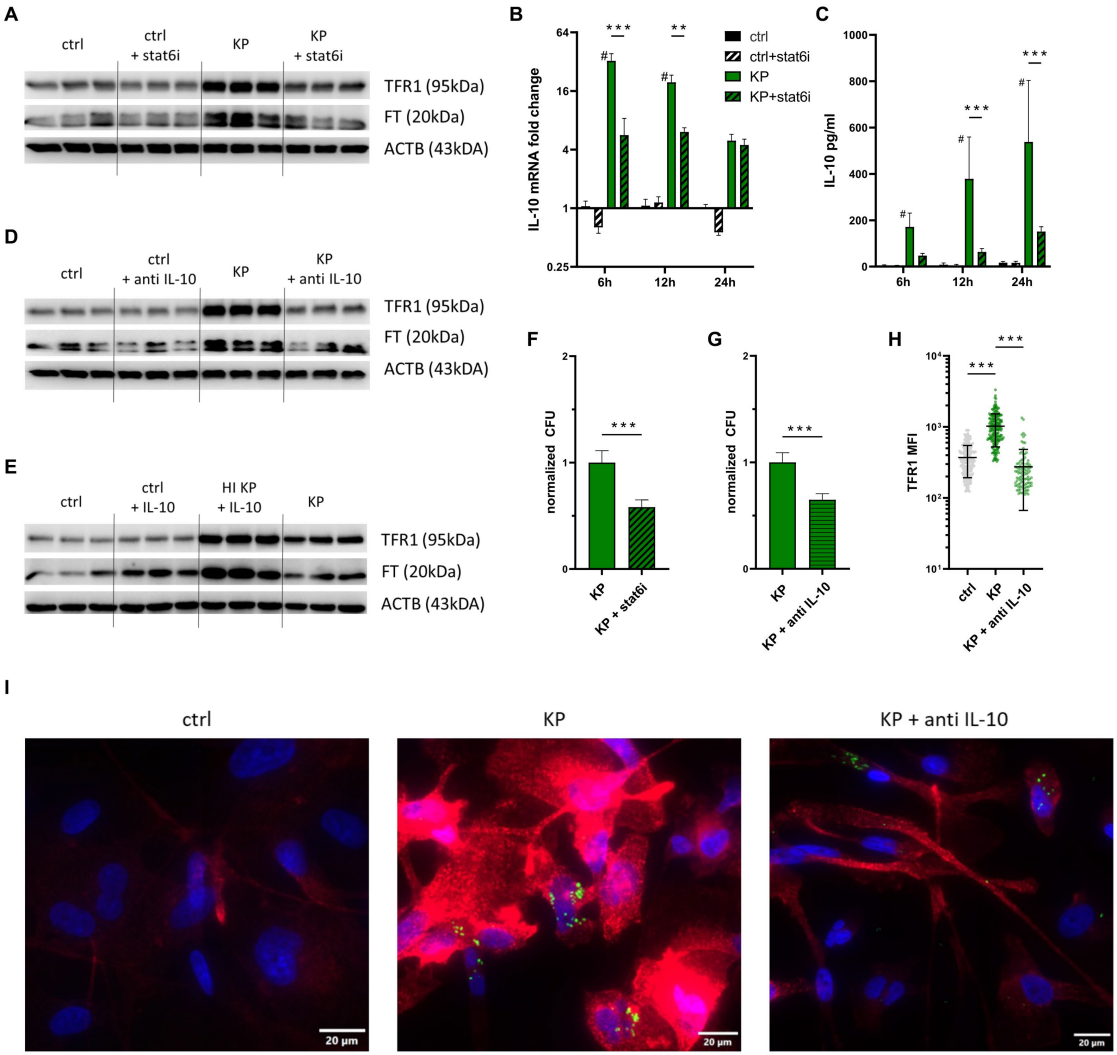
TFR1 induction is mediated by the STAT6-IL-10 axis. **(A)** TFR1 induction in infected cells is STAT-6 dependent. PMA-differentiated THP-1 cells were infected with KP for 1 h at MOI of 10. During the 24 h gentamicin protected, intracellular infection phase, cells were treated with 1 μM of the STAT-6-inhibitor (AS1517499) and subsequently harvested for Western blotting. Representative blot of three separate experiments. **(B)** Differential IL-10 mRNA expression of KP infected cells treated with STAT-6 inhibitor. Infected cells and uninfected controls were harvested at indicated time intervals of intracellular, gentamicin protected infection. Data shown as mean ± SEM of two separate experiments. **(C)** IL-10 levels in supernatants of infected cells treated with STAT-6 inhibitor, as determined by ELISA. Supernatants of infected cells and uninfected controls were collected at indicated time intervals of intracellular, gentamicin protected infection. Data shown as mean ± SD of two separate experiments. **(D)** TFR1 induction in infected cells is IL-10 dependent. Infected cells were treated with 40 μg/mL anti-human IL-10 blocking antibody during the 24 h intracellular, gentamicin protected infection phase und subject to Western blotting. Representative blot of three separate experiments. **(E)** Western blot of the iron uptake protein TFR1 and the iron storage protein FT in cells treated with inactivated bacteria or 40 ng/mL recombinant human IL-10. Representative blot after 24 h of infection of two separate experiments. **(F)** Intracellular bacterial numbers are decreased in cells treated with STAT-6 inhibitor. PMA-differentiated THP-1 cells were infected with KP for 1 h at a MOI of 10 and treated with 1 μM STAT-6 inhibitor (AS1517499) during the 24 h gentamicin-protected, intracellular infection phase. Afterwards, bacteria-containing lysates were serially diluted and plated onto LB-agar plates for CFU quantification. Data shown as mean ± 95% CI of three separate experiments, normalized to control condition. **(G)** Intracellular bacterial numbers are decreased in cells treated with anti IL-10. Infected cells were treated with 40 μg/mL anti-human IL-10 blocking antibody during the 24 h gentamicin-protected, intracellular infection phase. Afterwards, bacteria-containing lysates were serially diluted and plated onto LB-agar plates for CFU quantification. Data shown as mean ± 95% CI of three separate experiments, normalized to isotype-treated control condition. **(H)** MFI of TFR1 in infected cells treated with 40 μg/mL anti-human IL-10 blocking antibody during the 24 h gentamicin-protected, intracellular infection phase. MFI was measured from at least 100 cells per condition using the automated CellSense software. Data shown as mean ± SD. **(I)** Representative immune fluorescence imaging revealing the effect of IL-10 blockade on TFR1 expression in infected cells after 24 h of infection. Representative images, showing Ypet expressing bacteria (green rods) in cells stained for TFR1 (red) and nuclei (blue) at 400x magnification with a 20 μM scale bar. ^#^ denotes *p* < 0.05 compared to untreated controls, ^**^ denotes *p* < 0.01, ^***^ denotes *p* < 0.001 for *post-hoc* statistical testing. KP, *Klebsiella pneumoniae*; ctrl, control; stat6i, STAT-6 inhibitor; IL-10, interleukin 10; CFU, colony forming units; HI, heat-inactivated; TFR1, transferrin-receptor-1; FT, ferritin; ACTB, β-actin.

Next, we investigated possible mechanisms underlying increased TFR1-mediated iron uptake in KP infected macrophages. As recent work proposed the STAT6-IL-10-axis to be involved in KP-driven metabolic reprogramming, we investigated the effects of specific pharmacological inhibitors of this pathway (Dumigan et al., 2022). First, we stimulated macrophages with the STAT-6 inhibitor (AS1517499). Interestingly, the KP dependent TFR1 induction could be reduced, as analyzed by Western Blot (Figure 3A; Supplementary Figure S1). Protein levels of FT concomitantly were decreased. Next, we analyzed the cellular expression of the anti-inflammatory cytokine IL-10 over the course of infection (Figure 3B). IL-10 expression was significantly increased in cells infected with KP compared to uninfected cells, and treatment with the STAT-6 inhibitor during infection significantly diminished IL-10 expression in KP infected cells. To follow this up, we performed IL-10 ELISA of cell culture supernatants. In line with gene expression data, IL-10 was detected in high amounts in supernatants of infected cells, whereas treatment with the STAT-6 inhibitor substantially decreased IL-10 concentrations in supernatants (Figure 3C). As IL-10 emerged as a crucial mediator of KP induced metabolic changes, but is also known to regulate TFR1 expression in inflammatory macrophages (Ludwiczek et al., 2003), we applied a specific IL-10 blocking antibody during infection experiments. In agreement with our previous observations, IL-10 neutralization antagonized KP induced TFR1 induction, whereas the effect on FT induction was less pronounced (Figure 3D; Supplementary Figure S1). Interestingly, treatment of uninfected cells with recombinant human IL-10 did not lead to increased TFR1 levels (Figure 3E). In contrast, if cells were treated with inactivated bacteria together with recombinant IL-10, TFR1 was markedly induced, which is in line with previous observations on TFR1 regulation in macrophages (Tilg et al., 2002; Ludwiczek et al., 2003).

To quantify the effects of both specific inhibitors (STAT-6-inhibition and anti IL-10) on intracellular pathogen survival, we quantified intracellular bacteria after 24 h of infection. STAT-6 inhibition (Figure 3F), as well as IL-10 blockade (Figure 3G) led to significantly decreased numbers of intracellular CFUs. To exclude a direct bactericidal effect of the STAT-6 inhibitor AS1517499, a bacterial growth assay was performed (Supplementary Figure S3). This indicated that the STAT-6 inhibitor did not negatively affect the growth of KP *in-vitro*. We then applied immunofluorescence staining of cells to corroborate our findings. For this, cells were infected with KP carrying the Ypet plasmid (green) and stained for TFR1 (red). Expression of TFR1 was again increased in infected cells compared to uninfected controls, as measured in at least 100 cells per condition using automated CellSense software (Figure 3H). Treatment with a neutralizing IL-10 antibody significantly decreased TFR1 levels. Intracellular bacteria, as well as TFR1 expression patterns are also visible in provided immunofluorescence images (Figure 3I).

In sum, our data indicates that KP induces TFR1 expression in macrophages *via* the STAT-6-IL-10 axis to ensure a sufficient supply of iron for intracellular multiplication. Of note, inhibition of the STAT-6-IL-10 pathway results in reduced TFR1 expression and leads to impaired survival of KP within macrophages.

### 3.4. Inhibition of the STAT6-IL-10 axis leads to bacterial iron starvation

**Figure 4:**
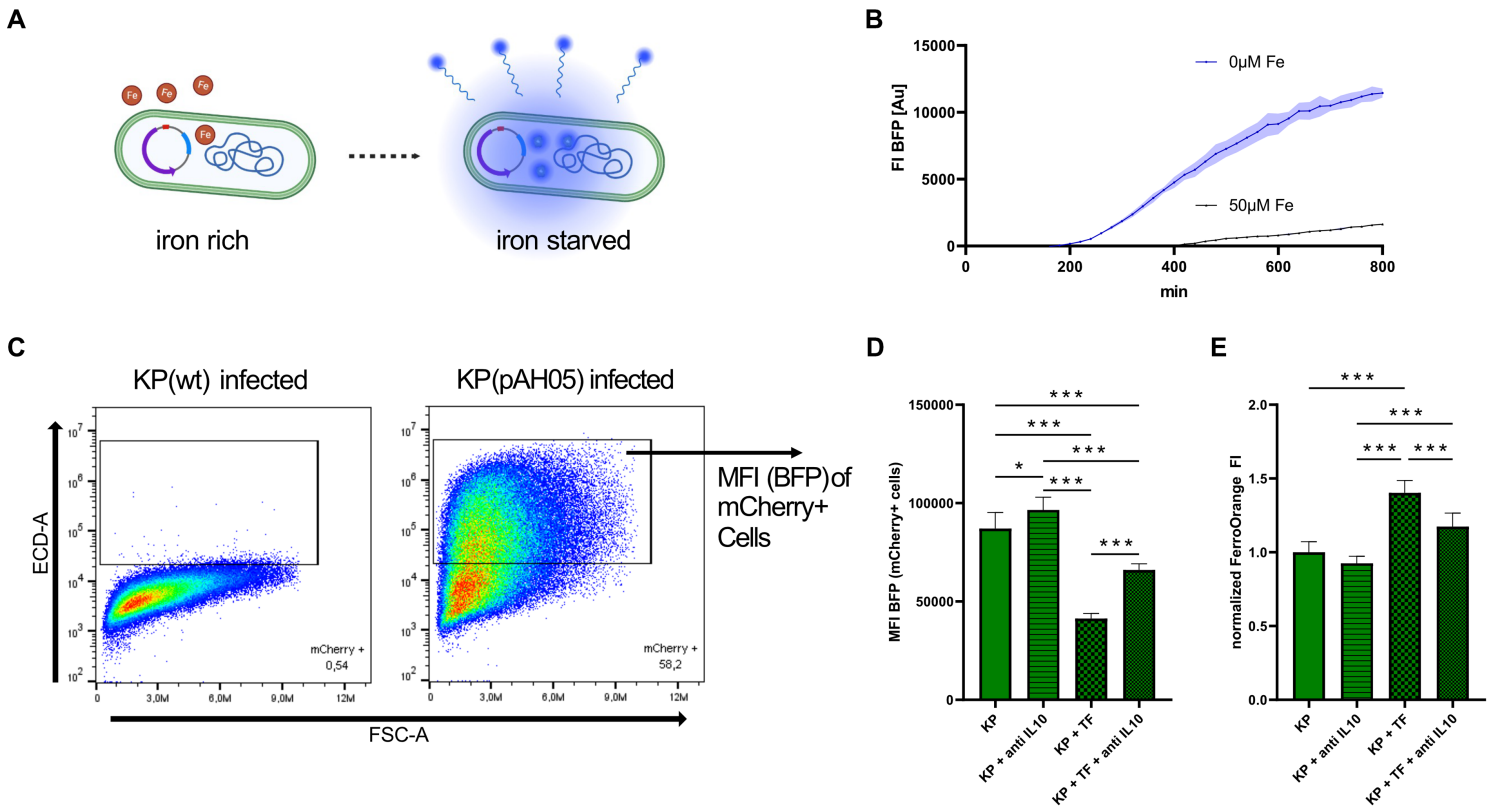
Inhibition of STAT6-IL-10 axis leads to bacterial iron starvation. **(A)** Illustrative image of bacteria containing the pAH05 iron-sensing plasmid. Bacterial expression of BFP is dependent on iron availability (RhyB2-promotor). Created with Biorender.com. **(B)** FI of BFP in bacterial cultures under iron starved and iron sufficient conditions. Bacteria containing the pAH05 plasmid were grown in LB to OD_600_ 0.5 and afterwards diluted to OD_600_ of 0.005 in iron free media (IMDM) or media supplemented with 50 μM iron (III) nitrate nonahydrate and immediately incubated in an automated microplate reader where FI of BFP was acquired every 15 min. Data shown as mean with ± SD as colored error bands. **(C)** Gating strategy of cells infected with bacteria containing the pAH05 iron-sensing plasmid. PMA-differentiated THP-1 cells were infected with KP (pAH05) for 1 h at MOI of 10 and followed by a 24 h gentamicin-protected, intracellular infection phase, and afterwards subject to flow cytometry analysis. Infected cells were identified as mCherry+. **(D)** MFI of BFP, as marker for iron starvation in intracellular bacteria, measured in infected (mCherry+) cells. Data shown as mean ± SD of three separate experiments. **(E)** Measurement of cellular iron levels *via* the fluorescent probe FerroOrange. Cells underwent 24 h gentamicin-protected KP infection and treatment with 50 μg/mL iron loaded TF or 40 μg/mL anti-human IL-10 blocking antibody. Data from three separate experiments shown as mean ± SD, normalized to KP infected cells. ^*^ denotes *p* < 0.05, ^***^ denotes *p* < 0.001 for *post-hoc* statistical testing. KP, *Klebsiella pneumoniae*; BFP, blue fluorescent protein; TF, transferrin; MFI, median fluorescent intensity; FI, fluorescent intensity; IL-10, interleukin 10.

To explore, whether inhibition of the STAT-6-IL-10 axis leads to growth limitation because of bacterial iron starvation, we employed flow cytometry of cells infected with KP containing the pAH05 plasmid, which encodes for iron sensitive promoters coupled to the expression of fluorescent proteins (NCBI OQ979407). As such, bacteria constitutively express mCherry (PybaJ promotor), GFP under high iron (SodB promotor) and BFP under iron starved (RhyB2 promotor) conditions (Figure 4A shows illustration, Figure 4B shows BFP fluorescence of bacterial cultures, Supplementary Figure S4 shows mCherry and GFP fluorescence of bacterial cultures). Using this approach, we determined the fluorescence of BFP, indicative of iron starvation, in infected (mCherry+) cells (Figure 4C) *via* flow cytometry. As the intracellular environment does not expose bacteria to excessive amounts of iron, GFP fluorescence of infected cells was not changed in any of the analyzed conditions (data not shown). Nevertheless, BFP mediated fluorescence was increased when infected cells were treated with anti IL-10, indicating iron limitation for bacteria. More prominently, treatment of infected cells with iron loaded TF reduced BFP expression, and this effect was largely reversed upon IL-10 neutralization (Figure 4D). Finally, we performed measurement of cellular iron content *via* FO under the same conditions (Figure 4E). In agreement with BFP-mediated indication of iron availability for bacteria, cellular iron levels were increased in infected and TF treated cells, whereas cellular iron content was markedly reduced upon anti-IL-10 treatment of KP infected macrophages.

Taken together, our results demonstrate that activation of the STAT-6-IL-10 axis during infection leads to increased iron supply to intracellular bacteria, and inhibition of this pathway reduces TFR1 mediated iron uptake and elicits iron starvation of intramacrophage bacteria, thereby diminishing their survival.

## 4. Discussion

Macrophages are at the center of innate immunity, and indispensable for early defense against bacteria (Weiss and Schaible, 2015). Specifically during KP infection, macrophages are critical players to limit KP outgrowth and to contain the infection (Broug-Holub et al., 1997; Hoh et al., 2019). Factors pivoting the balance of the KP-macrophage interaction in favor of the pathogen crucially affect host control of bacterial multiplication and thus infection outcome. One such factor is the availability of the nutrient iron which is essential for multiple metabolic functions of both, bacteria and eukaryotic immune cells (Andrews et al., 2003; Weiss and Schaible, 2015). Confined to the intra-macrophage space, KP is dependent on cellular iron acquisition to sustain growth. In this study, we provide evidence that viable but not inactivated KP induces metabolic changes in infected macrophages, which lead to increased cellular iron uptake *via* the STAT6-IL-10-TFR1 axis. Subsequent increased availability of this trace metal enforces pathogen persistence within macrophages.

Bacterial pathogens employ a wide array of strategies to overcome the antimicrobial activity of macrophages, including reprogramming of metabolic processes and arrest of phagolysosomal maturation (Flannagan et al., 2009; Weiss and Schaible, 2015). Both of these have been described in KP (Cano et al., 2015; Dumigan et al., 2022; Feriotti et al., 2022; Wong Fok Lung et al., 2022). Herein, we provide experimental evidence for a novel pathway by which KP enhances intracellular access to its essential nutrient iron. Specifically, KP increases the expression of the major iron uptake protein TFR1 in infected macrophages, thereby promoting uptake of TF bound iron, which becomes accessible for intracellular bacteria and contributes to their intracellular survival. Of note, when bacteria were opsonized with human serum prior to infection, TFR1 induction following infection was still observed (Supplementary Figure S5). In contrast, inactivated bacteria were unable to induce cellular TFR1, strengthening the hypothesis that this alteration in cellular iron trafficking is driven by soluble factors originating from or being induced by viable bacteria. Several bacterial pathogens have been reported to manipulate cellular iron metabolism in order to increase the availability of this nutrient during infection. Specifically in regard to iron uptake, *Ehrlichia*, *Coxiella*, and *Francisella* have been described to induce TFR1, while the intracellular pathogens *Salmonella* or *Listeria* do not (Barnewall et al., 1999; Howe and Mallavia, 1999; Nairz et al., 2007; Pan et al., 2010; Haschka et al., 2015). Our experiments revealed that the expression of the main cellular iron storage protein FT is increased in macrophages following bacterial infection but also upon stimulation with inactivated bacteria. This indicates that a pathogen associated molecular pattern mediated inflammatory response is responsible for ferritin induction and cellular iron scavenging (Torti and Torti, 2002; Haschka et al., 2021; Gehrer et al., 2023). In support of this hypothesis, macrophages treated with sterile LPS induce FT protein, but not TFR1 levels, in line with literature (Supplementary Figure S6) (Ludwiczek et al., 2003; Pandur et al., 2021).

Of note, the majority of our findings were experimentally shown in differentiated THP-1 cells. This cell line is widely used as a model for human macrophages, as once differentiated, cells closely mimic macrophage morphology, surface markers, cytokine production and infection control (Meijer et al., 2015; Starr et al., 2018). Nevertheless, our main finding, the induction of TFR1 during KP infection, was confirmed also in primary cells (Supplementary Figure S7).

High iron availability in the pathogen’s environment has been reported to promote not only KP growth but also virulence (Chen et al., 2020). Accordingly, exogenous stimulation with iron-loaded transferrin increased cellular iron content and bolstered intracellular KP survival. As KP is known to reprogram macrophage metabolism, these findings tempt us to speculate that KP is the main driver of the increase in cellular TFR1-mediated iron uptake. Recent work has uncovered that KP hijacks the STAT-6-IL-10 pathway to skew macrophages into a more pathogen-permissive state (Dumigan et al., 2022). Interestingly, specific pharmacological inhibitors of this pathway thwarted the KP-mediated TFR1 induction in our infection model. Accordingly, inhibition of this pathway led to decreased bacterial load, which was linked to limitation of iron availability to intracellular KP.

Mechanistically, KP infection of macrophages induces the expression of the anti-inflammatory cytokine IL-10. Clinical evidence suggests that treatment with IL-10 leads to iron sequestration by macrophages *via* stimulation of TFR1 and FT expression by both, transcriptional and posttranscriptional mechanisms (Tilg et al., 2002). Besides IL-10, other anti-inflammatory cytokines such as IL-4 and IL-13 similarly enhance iron uptake and storage in activated macrophages *via* increased TFR1 expression (Weiss et al., 1997). In contrast, in macrophages treated with solely inflammatory stimuli (interferon-γ and LPS), TFR1 levels and iron uptake are reduced, whereas combined treatment with IL-10 reversed these effects and stimulated TFR1-mediated iron acquisition (Byrd and Horwitz, 1993; Ludwiczek et al., 2003).

Adding another layer of complexity, excessive expression and secretion of IL-10, as reported during KP infection, may attenuate host defense (Yoshida et al., 2001; Peñaloza et al., 2016; Feriotti et al., 2022). This cytokine affects macrophage metabolism, inhibits antimicrobial effector mechanisms and is associated with a permissive environment for intracellular pathogens (Bogdan and Nathan, 1993; Peñaloza et al., 2016; Ip et al., 2017). Interestingly, numerous pathogens, including bacteria but also parasitic pathogens seem to be able to induce IL-10 during infection, thus pivoting the host-pathogen interaction in their favor (Redpath et al., 2001; Guha et al., 2021). In this regard, IL-10 blockade has been demonstrated to beneficially affect host immune functions and infection outcome against several intracellular pathogens (Cyktor and Turner, 2011). In line, the neutralization of IL-10 leads to better host survival in a murine KP-pneumonia model (Greenberger et al., 1995). In support of these findings, a later study evidenced elevated bacterial clearance and blocked KP dissemination in IL-10 knockout- compared to wildtype mice (Poe et al., 2013). In humans, polymorphisms related to a higher expression of the IL-10 gene are associated with higher severity in community-acquired pneumonia, although the detailed etiology was not studied (Gallagher et al., 2003).

The immune suppressive effects elicited by KP are considered to be capsule dependent (Yoshida et al., 2001; Dumigan et al., 2022). Interestingly, in our experiments, bacteria inactivated by either heat or antibiotic treatment were not able to increase macrophage TFR1. The addition of exogenous IL-10 combined with inactivated bacteria leads to TFR1 induction, similar as seen in cells infected with the viable pathogen. This suggests that a virulence or metabolic factor introduced only by the viable intracellular pathogen is responsible for macrophage reprogramming. Future studies are needed to fully uncover the major governing factors of the KP-macrophage interaction. Furthermore, the bacterial induced alterations in human iron metabolism identified herein are potentially therapeutically targetable, which should be evaluated in *in-vivo* studies, aimed to improve treatment of facultative intracellular bacteria in an era of exaggerating antibiotic resistance.

In summary, we provide evidence that KP is able to reprogram macrophage iron metabolism, in order to acquire sufficient amounts of this essential nutrient and to enforce intracellular persistence. KP is emerging as a notoriously resistant pathogen and global health threat (Effah et al., 2020; GBD Antimicrobial Resistance Collaborators, 2022). Insights into immune metabolism and its effects on this host-pathogen interaction will aid the development of novel antimicrobial therapies.

## Data availability statement

The raw data supporting the conclusions of this article will be made available by the authors, without undue reservation.

## Author contributions

PG, GW, MN, and IT planned and designed the project. PG, RH, CG, MG, NB, MS, and SB performed experiments. PG did the visualization of the data and performed the statistical analysis. AH designed, cloned, and verified the pAH05 plasmid. PG, GW, and MN prepared and created the initial draft. RH, CG, MG, IT, AH, and NB were included in the critical review and writing of the manuscript. GW, MN, and IT were responsible for supervision and funding acquisition. All authors contributed to the article and approved the submitted version.

## Funding

This project was enabled and supported by grants of the Austrian Science Fund (FWF, DOC 82 doc.fund; doctoral program MCBD and FWF, project P33062). Financial support by the Christian Doppler Society (Laboratory of iron metabolism and anemia research) and the “Verein zur Förderung von Forschung und Weiterbildung in Infektiologie und Immunologie an der Medizinischen Universität Innsbruck” is gratefully acknowledged.

## Conflict of interest

The authors declare that the research was conducted in the absence of any commercial or financial relationships that could be construed as a potential conflict of interest.

## Publisher’s note

All claims expressed in this article are solely those of the authors and do not necessarily represent those of their affiliated organizations, or those of the publisher, the editors and the reviewers. Any product that may be evaluated in this article, or claim that may be made by its manufacturer, is not guaranteed or endorsed by the publisher.

## Figures and Tables

**Table:** 

μg	microgram
μM	micromolar
μm	micrometer
ACTB	actin beta
BFP	blue fluorescence protein
CFU	colony forming units
CPS	capsular polysaccharide
FBS	fetal bovine serum
Fig	figure
FO	FerroOrange
FPN	ferroportin
FT	ferritin
GFP	green fluorescent protein
GM KP	gentamicin-killed *Klebsiella pneumoniae*
h	hour
HI KP	heat-inactivated *Klebsiella pneumoniae*
IL	interleukin
KP	*Klebsiella pneumoniae*
LB	lysogeny broth
LPS	Lipopolysaccharide
MFI	median fluorescence intensity
min	minute
MOI	multiplicity of infection
OD_600_	optical density at 600 nanometer
PMA	Phorbol-12-myristate-13-acetate
qPCR	quantitative real time polymerase chain reaction
STAT-6	signal transducer and activator of transcription – 6
STAT-6i	STAT-6 inhibitor
TFR1	transferrin-receptor-1

## Supplementary material

Supplementary Figure S1 Densitometry of protein expression of the transferrin receptor (TFR1) and ferritin (FT) relative to loading control β-actin accessed by Western Blots as presented in Figure 1B **(A)**, Figure 2B **(B)**, Figure 3A **(C)**, Figure 3D **(D)**, Figure 3E **(E)**. Data were normalized to controls and shown as mean ± SD. * denotes *p*<0.05, *** denotes *p*<0.001 for post-hoc statistical testing. KP, *Klebsiella pneumoniae*; ctrl, control; TFR1, transferrin-receptor-1; FT, ferritin; HI, heat-inactivated; GM, gentamicin-killed; IL-10, interleukin 10.

Supplementary Figure S2 Western blot of the iron uptake protein TFR1 and the iron storage protein FT in KP infected cells over the course of 24h intracellular infection. PMA-differentiated THP-1 cells were infected with KP for 1h at MOI of 10 and afterwards incubated for indicated time intervals in a gentamicin-protected, intracellular infection phase, before being subject to Western blotting. Representative blot from 2 separate experiments shown. KP, *Klebsiella pneumoniae*; ctrl, control; TFR1, transferrin-receptor-1; FT, ferritin; ACTB, β-actin.

Supplementary Figure S3 Direct impact of iron loaded TF and the STAT-6 inhibitor (AS1517499) on bacterial growth. **(A)** KP was grown in LB to OD_600_ 0.5, afterwards diluted to OD_600_ of 0.005 in cell culture media containing either 50µg/ml TF or 1µM STAT-6 inhibitor and immediately incubated in an automated microplate reader where OD_600_ was measured every 20min. Data shown as mean with ± 95%CI as colored error bands. **(B)** OD_600_ at 300min of bacterial growth. Data shown as mean with ± 95%CI. *** denotes *p*<0.001 for statistical testing. KP, *Klebsiella pneumoniae*; TF, transferrin; stat6i, STAT-6 inhibitor.

Supplementary Figure S4 OD_600_, mCherry and GFP fluorescence in cultures of KP (pAH05) during iron starved and iron sufficient conditions. Bacteria containing the pAH05 plasmid were grown in LB to OD_600_ 0.5 and afterwards diluted to OD_600_ of 0.005 in iron free media (IMDM) or media supplemented with 50µM iron (III) nitrate nonahydrate and immediately incubated in an automated microplate reader where OD_600_ **(A)**, and FI of mCherry **(B)** and GFP **(C)** was acquired every 15min. Data shown as mean with ± SD as colored error bands.

Supplementary Figure S5 Western blot of the TFR1 expression in KP infected cells after 24h of infection. Bacteria were incubated in PBS or human serum from healthy donors for 1h at 4°C prior to infection. KP, *Klebsiella pneumoniae*; ctrl, control; TFR1, transferrin-receptor-1; ACTB, β-actin.

Supplementary Figure S6 Western blot of the TFR1 and FT protein expression in cells treated with a sterile inflammatory stimulus (50ng/ml LPS/IFNg) for 24h. ctrl, control; TFR1, transferrin-receptor-1; FT, ferritin; ACTB, β-actin; LPS, lipopolysaccharide; IFNg, interferon-gamma.

Supplementary Figure S7 Western blot of TFR1 and FT expression in KP infected primary macrophages after 24h of infection. PBMCs from healthy donors were isolated from buffy coats. Subsequently, adherent cells were differentiated with M-CSF for 7 days, before infection with KP for 24h, as described in the methods section. KP, *Klebsiella pneumoniae*; ctrl, control; TFR1, transferrin-receptor-1; FT, ferritin; ACTB, β-actin.
