# Supplementary figures and images for: Klebsiella pneumoniae manipulates human macrophages to acquire iron
Source: Front Microbiol. 2023 Aug 11;14:1223113. doi: 10.3389/fmicb.2023.1223113 (PMC10451090; doi:10.3389/fmicb.2023.1223113)

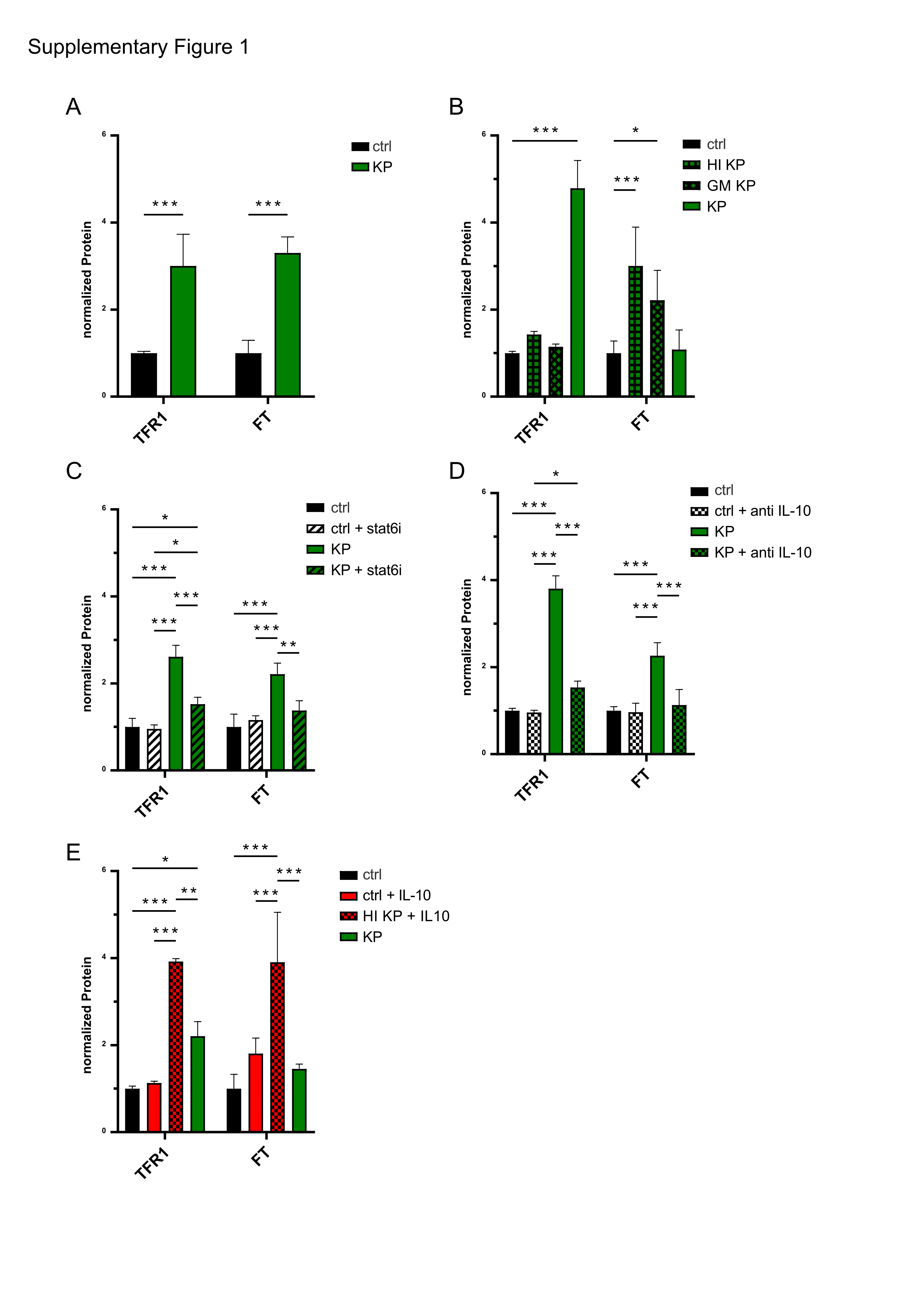

Supplement: Supplementary Figure S1 — Densitometry of protein expression of the transferrin receptor (TFR1) and ferritin (FT) relative to loading control β-actin accessed by Western Blots as presented in Figure 1B (A), Figure 2B (B), Figure 3A (C), Figure 3D (D), Figure 3E (E). Data were normalized to controls and shown as mean ± SD. * denotes p<0.05, *** denotes p<0.001 for post-hoc statistical testing. KP, Klebsiella pneumoniae; ctrl, control; TFR1, transferrin-receptor-1; FT, ferritin; HI, heat-inactivated; GM, gentamicin-killed; IL-10, interleukin 10. [file Image_1.TIFF]

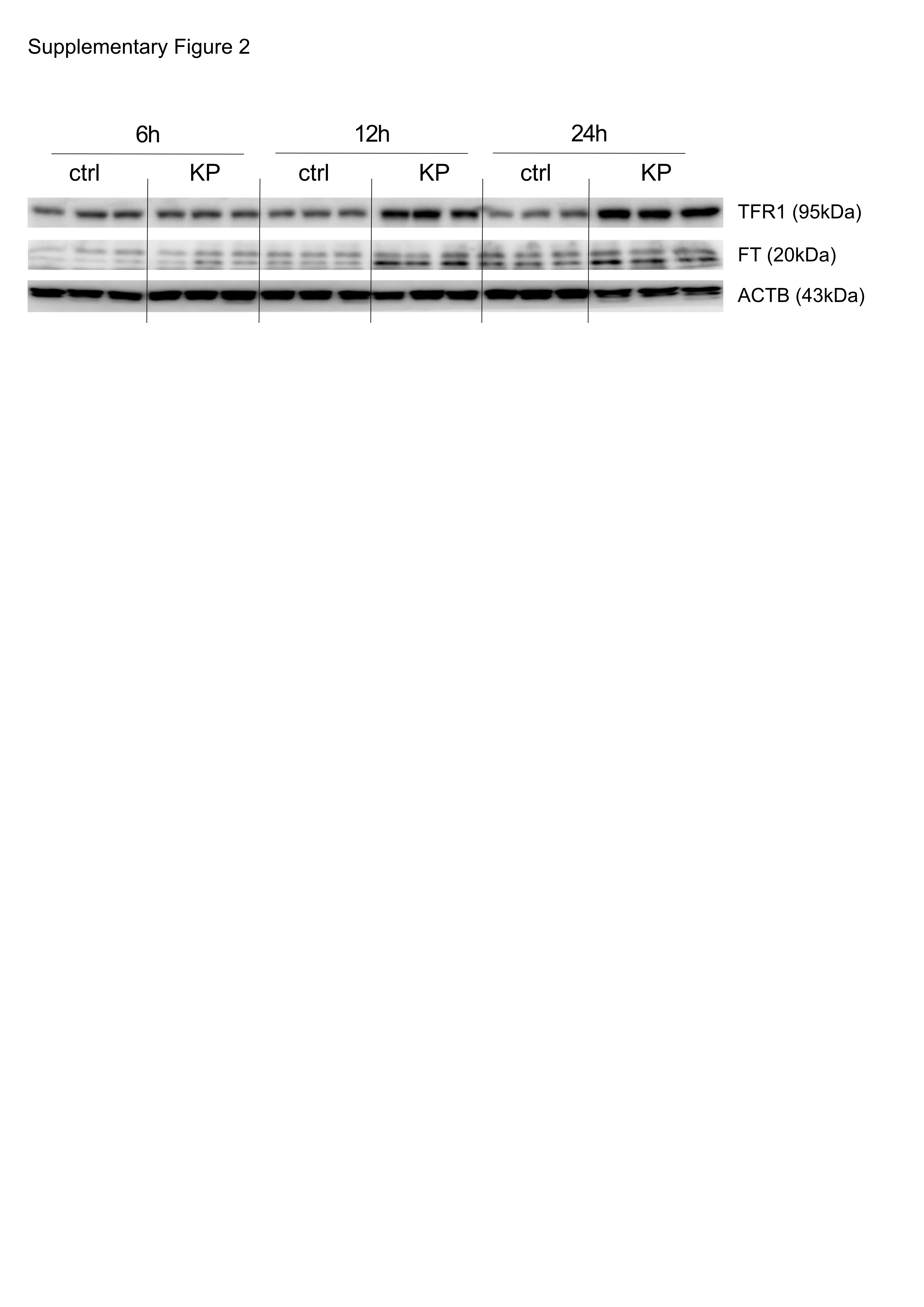

Supplement: Supplementary Figure S2 — Western blot of the iron uptake protein TFR1 and the iron storage protein FT in KP infected cells over the course of 24h intracellular infection. PMA-differentiated THP-1 cells were infected with KP for 1h at MOI of 10 and afterwards incubated for indicated time intervals in a gentamicin-protected, intracellular infection phase, before being subject to Western blotting. Representative blot from 2 separate experiments shown. KP, Klebsiella pneumoniae; ctrl, control; TFR1, transferrin-receptor-1; FT, ferritin; ACTB, β-actin. [file Image_2.TIFF]

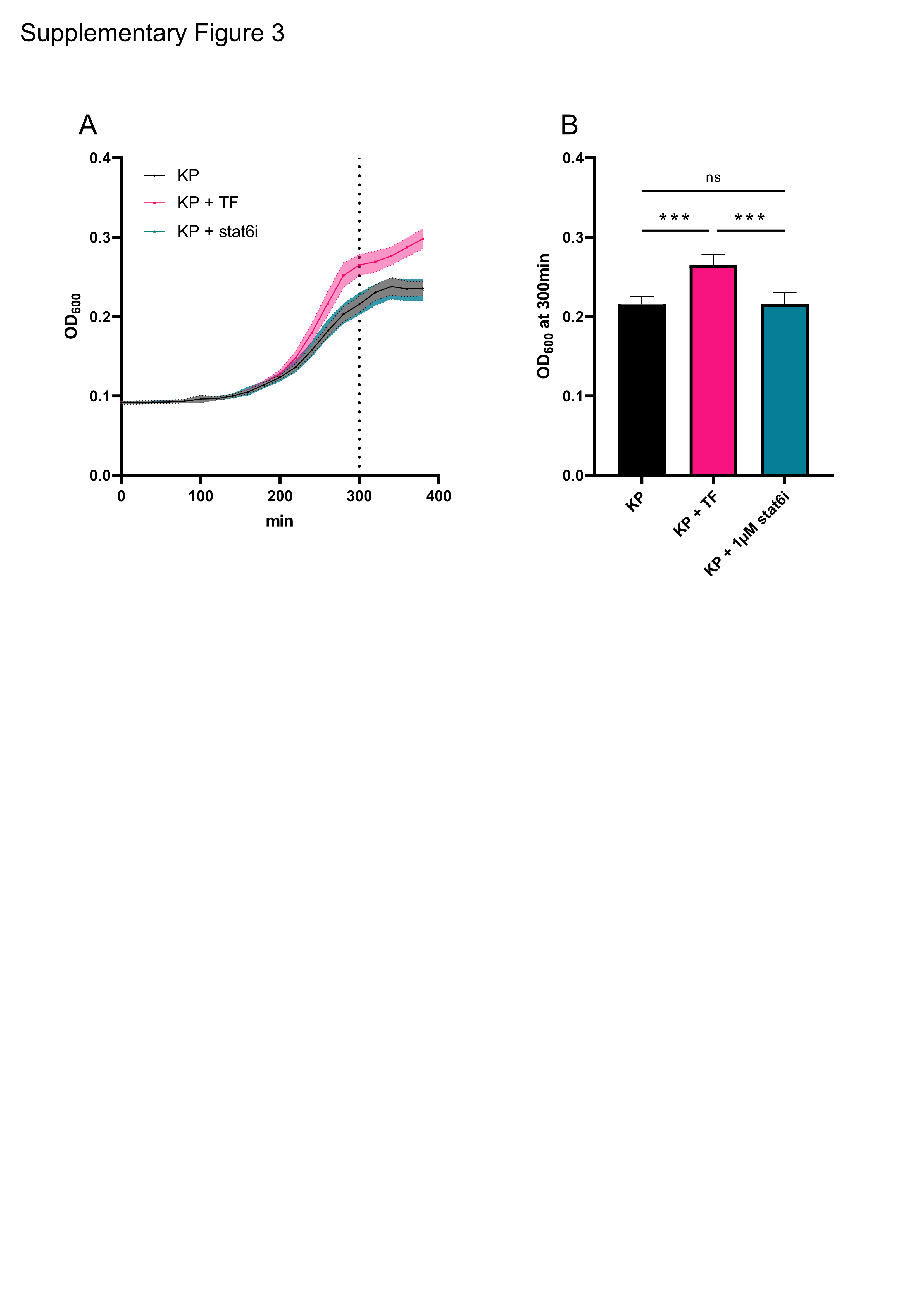

Supplement: Supplementary Figure S3 — Direct impact of iron loaded TF and the STAT-6 inhibitor (AS1517499) on bacterial growth. (A) KP was grown in LB to OD600 0.5, afterwards diluted to OD600 of 0.005 in cell culture media containing either 50µg/ml TF or 1µM STAT-6 inhibitor and immediately incubated in an automated microplate reader where OD600 was measured every 20min. Data shown as mean with ± 95%CI as colored error bands. (B) OD600 at 300min of bacterial growth. Data shown as mean with ± 95%CI. *** denotes p<0.001 for statistical testing. KP, Klebsiella pneumoniae; TF, transferrin; stat6i, STAT-6 inhibitor. [file Image_3.TIFF]

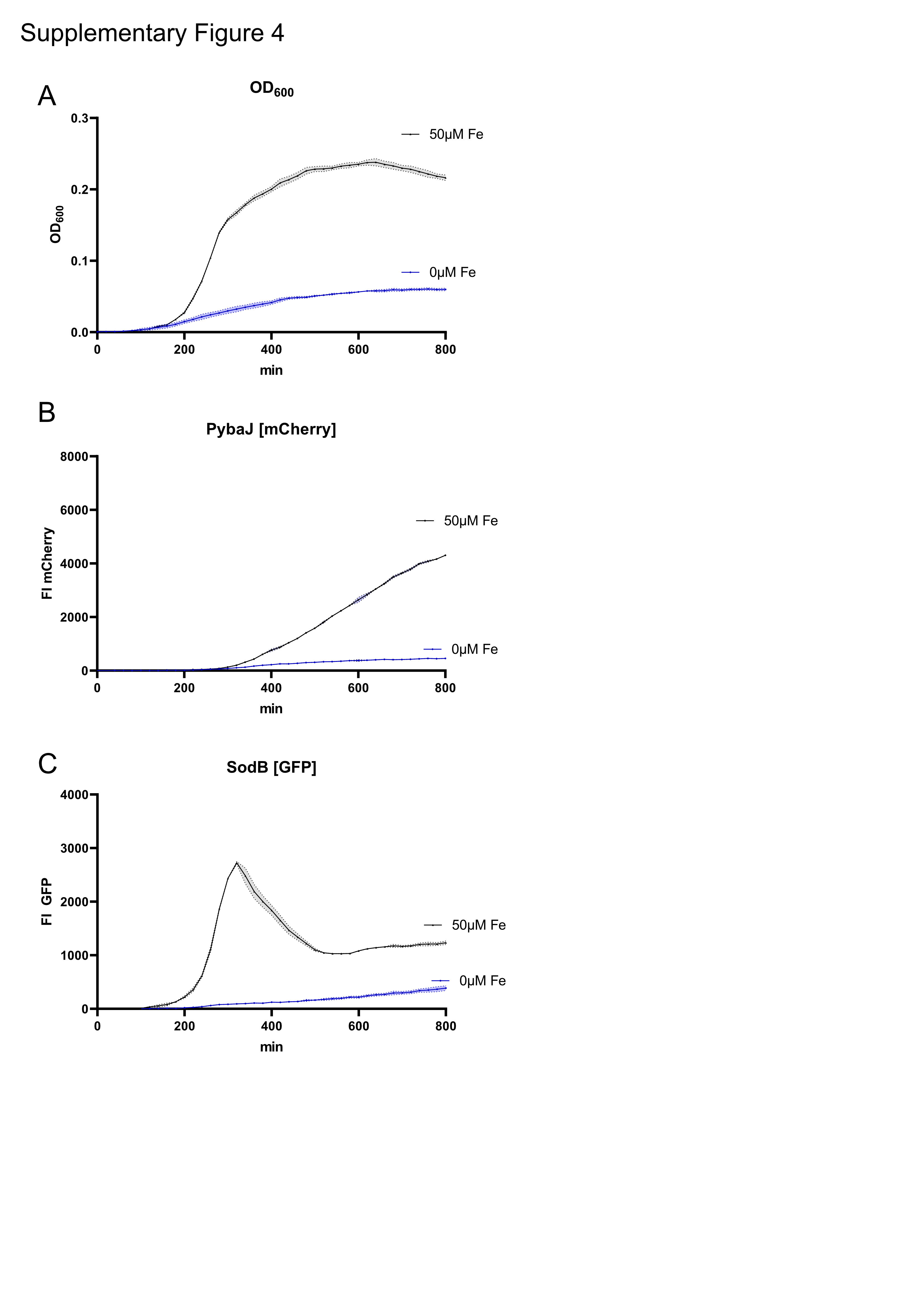

Supplement: Supplementary Figure S4 — OD600, mCherry and GFP fluorescence in cultures of KP (pAH05) during iron starved and iron sufficient conditions. Bacteria containing the pAH05 plasmid were grown in LB to OD600 0.5 and afterwards diluted to OD600 of 0.005 in iron free media (IMDM) or media supplemented with 50µM iron (III) nitrate nonahydrate and immediately incubated in an automated microplate reader where OD600 (A), and FI of mCherry (B) and GFP (C) was acquired every 15min. Data shown as mean with ± SD as colored error bands. [file Image_4.TIFF]

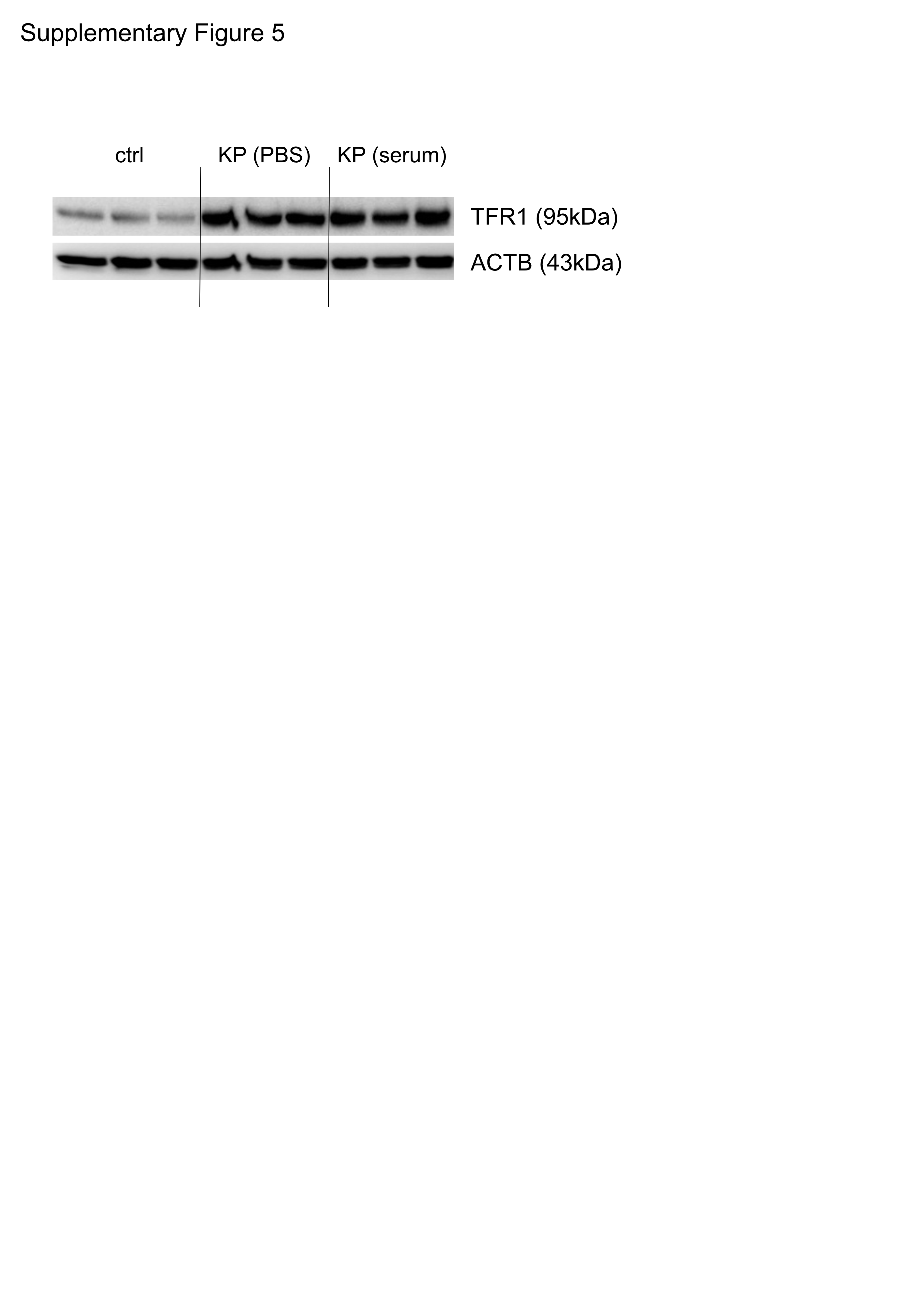

Supplement: Supplementary Figure S5 — Western blot of the TFR1 expression in KP infected cells after 24h of infection. Bacteria were incubated in PBS or human serum from healthy donors for 1h at 4°C prior to infection. KP, Klebsiella pneumoniae; ctrl, control; TFR1, transferrin-receptor-1; ACTB, β-actin. [file Image_5.TIFF]

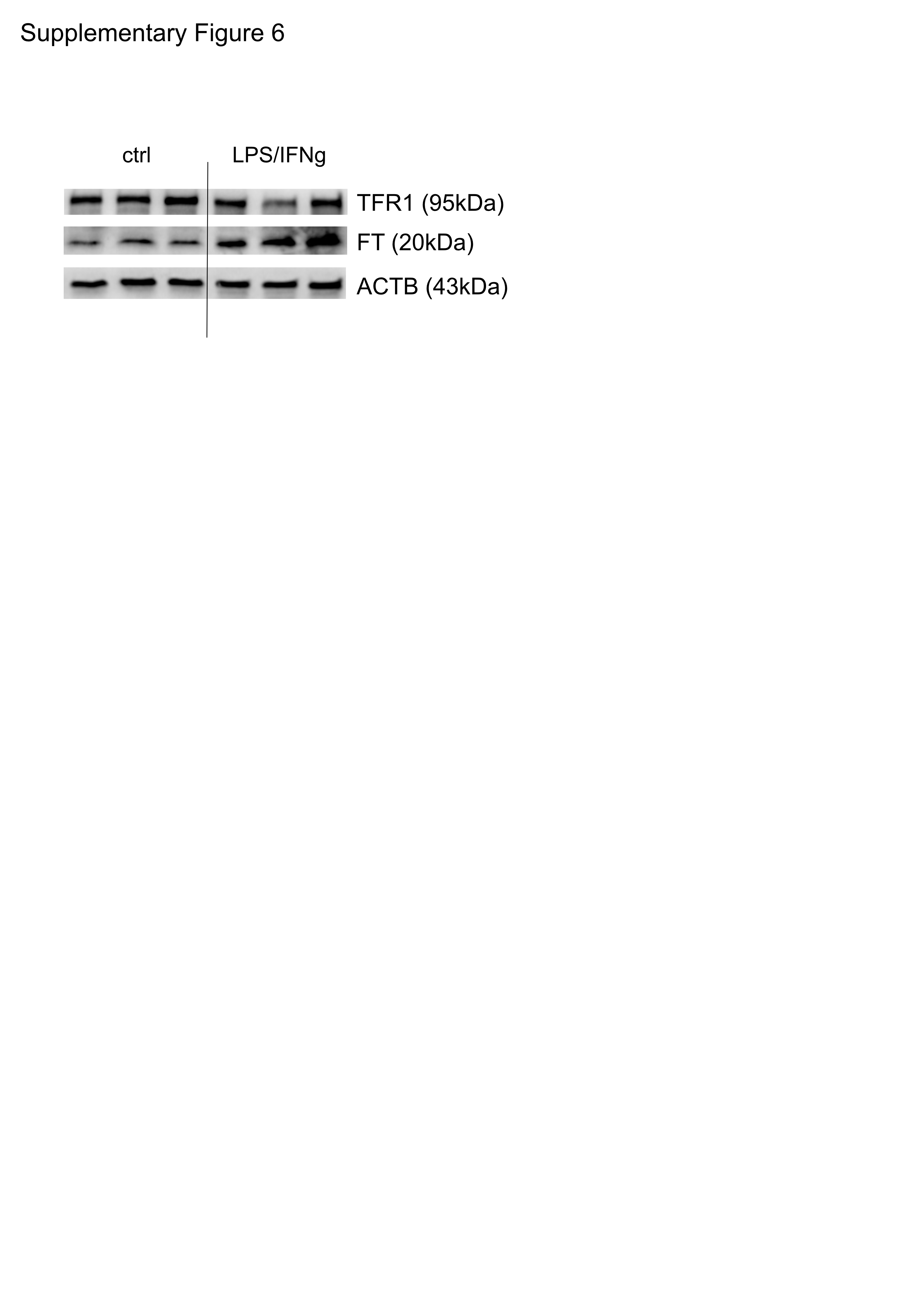

Supplement: Supplementary Figure S6 — Western blot of the TFR1 and FT protein expression in cells treated with a sterile inflammatory stimulus (50ng/ml LPS/IFNg) for 24h. ctrl, control; TFR1, transferrin-receptor-1; FT, ferritin; ACTB, β-actin; LPS, lipopolysaccharide; IFNg, interferon-gamma. [file Image_6.TIFF]

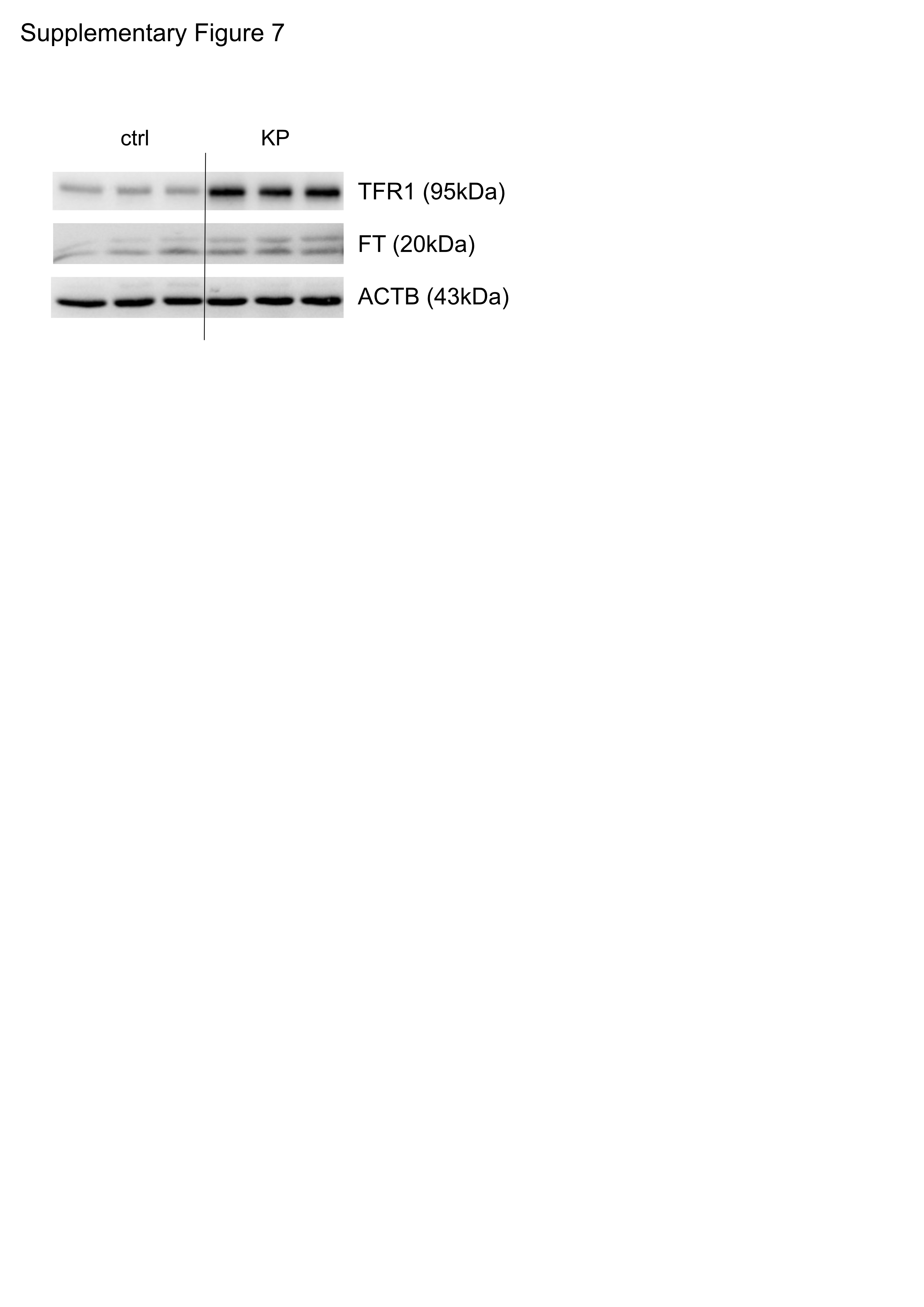

Supplement: Supplementary Figure S7 — Western blot of TFR1 and FT expression in KP infected primary macrophages after 24h of infection. PBMCs from healthy donors were isolated from buffy coats. Subsequently, adherent cells were differentiated with M-CSF for 7 days, before infection with KP for 24h, as described in the methods section. KP, Klebsiella pneumoniae; ctrl, control; TFR1, transferrin-receptor-1; FT, ferritin; ACTB, β-actin. [file Image_7.TIFF]
